# Homemade Modification of Salad Dressings to Reduce Their Erosive Potential

**DOI:** 10.3290/j.ohpd.b1993933

**Published:** 2021-09-11

**Authors:** Manuel J. Zoller, Alessio Procopio, Thomas Attin, Florian J. Wegehaupt

**Affiliations:** a Resident, Clinic for Conservative Dentistry and Preventive Dentistry, Center for Dental Medicine, University of Zürich, Zürich, Switzerland. Wrote the manuscript.; b Resident, Clinic for Conservative Dentistry and Preventive Dentistry, Center for Dental Medicine, University of Zürich, Zürich, Switzerland. Performed the experiments in partial fulfilment of requirements for a doctoral degree.; c Professor and Director, Clinic for Conservative Dentistry and Preventive Dentistry, Center for Dental Medicine, University of Zürich, Zürich Switzerland. Research idea, contributed substantially to discussion, proofread the manuscript.; d Head of Division of Preventive Dentistry and Oral Epidemiology, Clinic for Conservative Dentistry and Preventive Dentistry, Center for Dental Medicine, University of Zürich, Zürich, Switzerland. Research idea, hypothesis, experimental design, contributed substantially to discussion and writing the paper, proofread the manuscript.

**Keywords:** erosion, modifications, plain yoghurt, salad dressings, tooth wear

## Abstract

**Purpose:**

To investigate the possibility of reducing the erosive potential of salad dressings by adding yoghurt.

**Materials and Methods:**

Two hundred enamel samples from bovine teeth were allocated to 20 groups (n = 10). Three modified commercially available balsamic dressings (addition of 10%, 20%, 50% yoghurt or 8.8 mM calcium chloride) and two homemade salad dressings with and without modifications were tested. Enamel samples were eroded for 2 min, rinsed for 30 s with tap water and finally abraded (20 brushing strokes with toothpaste slurry). After 40 of these cycles of erosion/abrasion, the dental hard tissue loss was determined by contact profilometry.

**Results:**

For commercially available salad dressings, modification yielded a statistically significant decrease in enamel wear. The exception was Anna’s Best Dressing Balsamico modified with 8.8 mM calcium chloride, for which no reduction was found compared with the unmodified dressing. For all homemade dressings, a significant reduction was observed when modified with 20% yoghurt. However, when only 10% yoghurt was added to the homemade dressings, an increase of the erosive potential was observed compared to the unmodified dressing.

**Conclusions:**

The study shows that increasing the calcium concentration only with calcium chloride in commercially available salad dressings did not show predictable outcomes to reduce erosion. However, mixing 20% plain yoghurt into the dressings reduced the erosive potential statistically significantly.

In erosive tooth wear, no microorganisms are involved. Erosions are induced by either endogenous or exogenous organic or inorganic acids.^2,12,48,57^ Endogenous acids originate from the body itself and exogenous acids are supplied from the outside.

Intrinsic erosions are caused by stomach acid rising into the oral cavity.^25^ The reasons for this situation are diverse, such as eating disorders like anorexia nervosa and bulimia nervosa, alcohol abuse, gastroesophageal reflux disease and even pregnancy might be temporary causes for gastric acid reaching the oral cavity.^36,38^

On the other hand, exogenous acids have many origins. Soft drinks, fruit juices, alcoholic beverages and even medications contain these erosive acids.^8,22,31^

A systematic review by Chan et al^10^ showed that the role of dietary acids and habits in tooth erosion in adolescents needs to be studied more closely in order to establish more evidence-based conclusions. They reported the consumption of vinegar as a relevant factor resulting in erosive tooth wear. The consumption of acidic drinks has seen a sharp increase in recent years.^30,39,49^ A recent study by Salas et al^40^ found that repeated consumption of natural fruit juices, acidic snacks and sweets involves an increased risk of erosion. In contrast, the consumption of products such as milk and yoghurt are regarded as protective foods in terms of erosion development.^40^ It is important to consider that it is not just the amount of erosive food that plays a decisive role, but rather the frequency and how long a substance remains in the oral cavity. It was found that a daily consumption of more than four acidic units are very strongly associated with erosion.^34,37^ A previous study demonstrated that balsamic vinegar-based dressings (Italian-type) have a significantly higher erosive potential than orange juice. In contrast, dressings containing calcium-rich products (enriched with milk and/or cream) (French-type) caused less enamel wear than orange juice.^21^ Furthermore, it was shown that the erosive potential of an erosive beverage such as orange juice could be significantly reduced if modified with a effervescent calcium tablet.^50^ It was concluded that the increase of calcium content of the orange juice by adding the effervescent calcium tablet was responsible for the decreased erosive potential. Also, diets that were modified with minerals, such as calcium or phosphate and/or casein phosphopeptide-amorphous calcium phosphate (CPP-ACP), showed reduced erosivity.^49^

Salad dressings are complex mixtures of different ingredients, such as vinegar, oil and herbs. It would thus be interesting to determine whether these complex and erosive solutions could also be modified in terms of reduction of erosivity.

Therefore, the aim of the present study was to investigate whether erosive (commercially available and homemade) salad dressings can be modified to reduce their erosive potential. As this modification should be applicable by the consumers at home, a possible modification by simply adding plain white yoghurt was investigated. It has to be noted that yoghurt by itself is an acidic but not erosive foodstuff, because yoghurt is supersaturated with calcium and phosphate compared to the tooth enamel.^49^

The null hypothesis of this study was that there is no difference in the erosive tooth wear caused by unmodified and modified erosive salad dressings.

## Materials and Methods

### Preliminary Calculation

Based on the pH-value, the calcium content and the observed erosive tooth wear of the tested salad dressings in a previous study,^21^ a multiple linear regression model was developed, in which the erosive tooth wear was estimated by using the explanatory variables H^+^ and Ca^2+^ concentration. With this mathematical model, the amount of calcium required to minimise the erosive potential could be calculated. The coefficient of determination (R2) of the multiple linear regression (log_(enamel wear)_ = b_0_ + b_1_ * [H^+^] + b_2_ * [Ca^2+^] + b3 * [H^+^] * [Ca^2+^]) is 0.8287. This means that about 82% of the erosive enamel loss can be explained by the two variables proton concentration [H^+^] and calcium concentration.

In order to check this mathematical model, a calcium concentration of 8.8 mmol/l was calculated for all dressings, so that the resulting enamel loss would be < 1 μm.

### Sample Preparation

A total of 200 bovine enamel samples (n=200) were produced and randomly divided into 20 groups (G1-G20, n=10). Enamel samples with a diameter of 3 mm were obtained from bovine mandibular anterior teeth by using a diamond hollow drill (Proxxon, Brütsch/Rüegger Werkzeuge; Urdorf, Switzerland). Then, the samples were embedded in acrylic resin (Paladur, Heraeus Kulzer; Hanau, Germany) so that the final diameter was 6 mm. As a final step, the samples were polished to achieve a smooth surface. For this purpose, a grinding machine was used with sandpaper (GEKO SiC Foil, Struers; Ballerup, Denmark) with grain sizes of 1000 grit (10 s), 2000 grit (20 s) and 4000 grit (40 s) at a speed of 150 rpm under water cooling.

### Modification of Salad Dressings

The erosive potential of commercially available salad dressings used in this study (Anna’s Best Dressing Balsamico, Tradition Sauce Balsamico and M-Classic Dressing Italian) is known from a previous study by Hartz et al.^21^ Each dressing was mixed with 100 mM CaCl_2_ solution to reach a calcium concentration of 8.8 mM to test the mathematical model above.

Plain yoghurt (10%, 20% and 50%) was added to each of these three commercial available salad dressings. In order to obtain a comparison between homemade and commercially available dressings, two different homemade Italian dressings were prepared following the Intercantonal Teaching Aids for Housekeeping Lessons.^1^ The composition of the three commercially available and two homemade salad dressings is presented in Table 1. The two homemade dressings were also modified with 10% and 20% of plain yoghurt.

**Table 1:** Ingredients of commercially available and homemade salad dressings and modification substances

Product	Ingredients (manufacturer’s information)
Anna’s Best Dressing Balsamico*	Water, Aceto balsamico di Modena I.G.P. 30%, (red wine vinegar, grape must concentrate, colouring agent: E 150d), olive oil, sunflower oil, sugar, concentrated grape must, seasoning, saline, natural flavourings, thickener E 415
M-Classic Dressing Italian*	Water, red wine vinegar 37%, olive oil 7%, sugar, sunflower oil, table salt, spices (maltodextrin, sugar, oregano, parsley, chives, red pepper, garlic, pepper, table salt, sunflower oil, flavour enhancer: E 621), onion, elderberry concentrate, natural flavours, basil, parsley, thickeners E 415 and E 401
Tradition Sauce Balsamique*	Sunflower oil, Aceto balsamico di Modena 30% (wine vinegar, concentrated grape must),water, burnt sugar, table salt, concentrated lemon juice, yeast extract, garlic, spice, onion, thickener E 415, basil, natural flavour (contains celery), pepper
Tiptopf Italienische Salatsauce A / B (45% vinegar / 31% vinegar)	Salt or spice, pepper 2-3 tablespoons of red wine vinegar or balsamic vinegar 4-5 tablespoons oil (preferably olive) 1-2 garlic cloves
M-Classic red wine vinegar*	Red wine vinegar, antioxidant: potassium metabisulphite. Acidity 4.5%
M-Classic olive oil, cold-pressed*, made in Spain	Olive oil
Bio yoghurt nature*	Whole milk, skimmed milk powder, milk proteins

*Made for Migros Cooperative, Zürich, Switzerland.

The possible impact of viscosity was tested by modifying the two dressings with a hydroxyethyl-cellulose solution (Merck; Darmstadt, Germany) with Zürich tap water. This solution was used to replace olive oil in an amount that would yield the same viscosity as the dressings modified with plain yoghurt.

The viscosity was determined in each case using a viscometer (Becker Research Equipment; Göttingen, Germany) with the associated software (3.x, SynopsisLogic; Rosdorf, Germany).

### Erosive/Abrasive Procedure

The enamel samples were eroded in 3 ml of the respective salad dressings or their modifications for 2 min per sample under constant motion by gently shaking the container with the samples and the solution. After 2 min, the samples were rinsed with tap water for 30 s. The subsequent abrasion (20 brushing strokes, load 2 N) was carried out as described by Hartz et al.^21^ After brushing, the samples were again rinsed with tap water to remove remnants of the toothpaste slurry. A total of 40 cycles of erosion followed by toothbrush abrasion were performed for each sample. For each erosive and abrasive attack, fresh solutions and slurry were used.

### Determination of Enamel Wear

In order to determine the erosive/abrasive enamel wear, surface profiles of the sample were recorded at baseline using contact profilometry (MarSurf GD25, Mahr; Göttingen, Germany). Surface profilometry is described in detail by Hartz et al.^21^ Before starting the erosive/abrasive procedure, reference areas (areas outside two parallel scratches made on enamel and resin) were covered with adhesive tape, leaving a test area in between the reference areas uncovered.^21^

### Statistical Analysis

Values of enamel wear for the unmodified commercially available dressings were taken from the study by Hartz et al.^21^

In the first part, dental hard tissue losses of the unmodified and modified dressing groups were compared using the Kruskal-Wallis rank-sum test to check the independence of the data. In the next step, the multiple independent data were compared with each other by pairwise comparison using Conover’s post-hoc test. The significance level was set at p ≤ 0.05.

All statistical work, including the creation of the mathematical model to determine the ablation, was carried out with the statistical software R (R Foundation for Statistical Computing; Vienna, Austria) including the packages tidyverse and PMCMR.

## Results

The resulting enamel wear after 40 cycles of erosion and abrasion for the different dressings is presented in Table 2.

**Table 2:** Median and interquartile range of enamel wear [μm] for the unmodified and modified commercially available and homemade salad dressings

Product	Enamel wear in μm	
	median	interquartile range
**Commercial dressing: Anna’s Best Dressing Balsamico**		
*Anna’s Best Dressing Balsamico	5.0	1.8
Anna’s Best Dressing Balsamico 10% yoghurt	3.7	1.5
Anna’s Best Dressing Balsamico 20% yoghurt	0.9	0.8
Anna’s Best Dressing Balsamico 50% yoghurt	0.7	0.4
Anna’s Best Dressing Balsamico 8.8 mmol/l calcium	5.1	1.2
**Commercial dressing: Tradition Sauce Balsamique**		
*Tradition Sauce Balsamique	9.5	5.3
Tradition Sauce Balsamique 10% yoghurt	1.0	0.6
Tradition Sauce Balsamique 20% yoghurt	0.2	0.1
Tradition Sauce Balsamique 50% yoghurt	0.1	0.1
Tradition Sauce Balsamique 8.8 mmol/l Calcium	1.4	0.2
**Commercial dressing: M-Classic Dressing Italian**		
*M-Classic Dressing Italian	10.9	12.3
M-Classic Dressing Italian 10% yoghurt	7.2	0.8
M-Classic Dressing Italian 20% yoghurt	1.9	1.2
M-Classic Dressing Italian 50% yoghurt	0.2	0.05
M-Classic Dressing Italian 8.8 mmol/l calcium	7.8	3.0
**Homemade dressing: Tiptopf Italienische Salatsauce A**		
Tiptopf Italienische Salatsauce A	5.8	2.0
Tiptopf Italienische Salatsauce A + 10% yoghurt	13.1	4.2
Tiptopf Italienische Salatsauce A + 20% yoghurt	1.1	0.3
Hydroxyethyl-Cellulose 45% vinegar	14.8	1.9
**Homemade dressing: Tiptopf Italienische Salatsauce B**		
Tiptopf Italienische Salatsauce B	1.4	0.3
Tiptopf Italienische Salatsauce B + 10% yoghurt	5.7	1.2
Tiptopf Italienische Salatsauce B 20% yoghurt	0.4	0.2
Hydroxyethyl-Cellulose 31% vinegar	13.4	1.3

The values for dressings marked with * are taken from the study by Hartz et al.^21^ For all modified dressings, statistically significantly different enamel wear compared with the respective unmodified dressings was observed, except for Anna’s Best Dressing Balsamico modified with 8.8 mmol/l calcium.

Table 2 shows that the mathematical model for the desired reduction in erosive potential after the addition of calcium chloride could not be verified because the values were unpredictable and not less than the anticipated 1 μm.

Within the respective dressings, a significant difference between the unmodified dressings compared with the different modifications was observed (p ≤ 0.05). As the amount of plain yoghurt increases, a decrease in the erosive potential of the commercially available salad dressings was observed. However, the modification of Anna’s Best Dressing Balsamico with 8.8 mmol/l calcium resulted in no statistically significant reduction of the observed enamel wear.

For the homemade salad dressings, a minimum of 20% of yoghurt was needed to decrease the dental hard tissue loss. Homemade dressings with only 10% yoghurt showed an even higher erosive potential than the unmodified dressing itself.

## Discussion

The enamel samples were subjected to a total of 40 erosion and abrasion cycles. The erosion lasted 2 min per cycle and the abrasion was carried out with a constant applied weight of 2 N and 20 brush strokes. These correspond to the parameters used in other erosion/abrasion studies.^52^ All in all, the samples were eroded for 80 min and abrasion was induced with 800 brush strokes in total. The samples were stored in tap water between the cycles and overnight, which has no influence on the measurements.^3^ Conducting abrasion directly after the erosive attack should represent the worst-case scenario when a person brushes his/her teeth immediately after consumption of erosive foodstuff.^5^ One might assume that waiting after the erosive attack before toothbrushing might minimize the resulting tooth wear. However, numerous studies^5,15,47^ show that a mineralisation cycle between the erosive and abrasive attack has only a small effect on dental hard tissue loss. A recent systematic review and meta-analysis reported no statistically significant difference in the erosive tooth wear of human enamel between delayed and immediate toothbrushing, whereas significantly less erosive tooth wear of bovine enamel was observed after delayed toothbrushing.^24^ Therefore, they concluded that bovine and human teeth behaved differently in response to erosion and toothbrush abrasion. In the present study, toothbrushing abrasion of the bovine enamel was performed directly after the erosive attack (immediate toothbrushing); therefore, it can be assumed that the findings of the above mentioned systematic review do not apply to the results of the present study.

Contact profilometry is a popular method used in erosion and abrasion studies.^26,46,51^ If there are too few cycles, however, the physical measurement using contact profilometry reaches its limits, as only a few nanometers of dental hard tissue are lost per cycle. In order for contact profilometry to perceive a difference between the height profiles, at least 0.105 μm has to be removed from the sample surfaces.^3^ The disadvantages of profilometry are also possible scratches by the needle when scanning the samples. This can of course lead to a corruption of the values.^23,41^

Due to the high number of samples (n=200) required in this study, it was advantageous to use bovine teeth. Bovine teeth have a larger surface area than human teeth. This allowed the preparation of several samples per tooth, which further increases comparability of the samples.^52,54^ Moreover, bovine teeth do not have caries, which increases the homogeneity of the samples compared to human teeth.^35,56^ However, it is in fact the case that bovine enamel and human enamel differ in some parameters.^28^ Bovine enamel has a lower proportion of calcium and phosphate.^9,14^ This leads to faster demineralisation and can therefore clearly be seen as a disadvantage in an erosion study like this one.^42^ The loss of dental hard tissue is therefore assumed to be greater in studies with bovine enamel than it would be the case with human enamel.^6^ Since the original salad dressings were compared with the homemade modified dressings, the use of bovine enamel is acceptable, as only wear is compared within this study.

The null hypothesis of the present study, that there are no differences in the erosive tooth wear caused by the unmodified and modified erosive salad dressings, has to be rejected. For the commercial dressings, a clear reduction of the resulting erosive/abrasive enamel wear was observed when yoghurt (irrespective of the amount) was added.

Yoghurt as a household modification, with its acidic but calcium-rich ingredients,^49^ has proven to be very effective in reducing erosive potential. Another study^32^ confirms that erosivity does not depend exclusively on the respective pH-value of beverages and foodstuffs. Rather, saturation with minerals (Ca, P and F) compared to the dental hard tissue is responsible as the driving force for dissolution.^32^ Of course, adding yoghurt affects the consistency and taste of the salad dressings. The extent to which these changes disturb the consumer was not taken into account in this study. But it is precisely the influence of viscosity that should not be neglected as an influencing factor. In principle, a higher viscosity actually led to a lower erosive potential in spite of a constant acid concentration.^7^ This is due to the fact that the acid flow rate is reduced over the enamel and the adjacent Nernst layer over the dental hard tissue allows for less ion exchange.^4^ This possible effect was tested by modifying the homemade dressings with hydroxyethyl-cellulose as a thickener and oil substitute.

In contrast to the situation with the commercially available dressings, the addition of 10% yoghurt to the homemade dressings even led to an increase in the erosive/abrasive enamel wear. It is assumed that the yoghurt in smaller concentrations initially acts as an emulsifier and homogenizes the oil/vinegar mixture, which initially increases the erosive potential. This effect was not observed in commercially available dressings, since it must be assumed that they already contain various emulsifiers.

As erosions are often associated with low pH values, the immediate effect would be to ask for the pH values of the dressings used here. Both kinds of dressings (commercially available and homemade dressings) as well as the yoghurt are emulsions, but pH values can only be measured in the aqueous phase of solutions. This is especially important when the pH value of the homemade dressings are reported, as they only consist of vinegar and oil, with the vinegar as the aqueous phase. Since the same vinegar was used throughout the experiment, the same pH value would be measured in both homemade dressings; however, different enamel wear was observed for the two kinds of homemade dressings (median/IQR: 5.8/2.0 and 1.4/0.3). This result supports the finding that not just the pH of a solution, but also its saturation with respect to tooth minerals is the driving force behind erosive demineralisation.^42^

There are many other influencing factors, such as buffering capacity,^27^ temperature,^13,45^ and liquid flow rate,^43,53^ that were not taken into account in this in vitro study. Next to eating and drinking behaviour, biological factors such as salivary flow rate, saliva composition, and position of the teeth are also erosion-modifying factors.^33^ The saliva itself dilutes and neutralises acids and is therefore the most important biological factor protecting the dental hard tissues against erosion.^20,55^ The salivary flow rate is increased by stimulation even before an erosive food is consumed. The increased saliva flow rate increases the clearance and thus has an additional protective effect against erosion.^11,29^

The pellicle offers further physiological protection. It is a kind of semipermeable membrane which is made up of various proteins, peptides, lipids, and other biopolymers. The pellicle forms in the saliva and covers the teeth within minutes. It reduces the diffusion of calcium and phosphate out of the tooth structure and has a certain acid resistance itself, which protects the tooth from erosions.^16-19,44^

Thus it must be mentioned that, although this in vitro study might not completely reflect the true intraoral situation, it was nevertheless able to show reduced erosive action of the modificated salad dressings. The main focus was not to measure the absolute enamel wear caused by the salad dressings, but to determine whether the modifications have an influence on the erosive potential of the respective dressings. The study by Hartz et al^21^ shows the difference in the erosive potential between the French- and Italian-type dressings, and patient should be made aware of that difference

## Conclusion

This study showed that the addition of yoghurt to commercially available salad dressings statistically significantly reduced their erosive potential. In the case of the homemade dressings, however, it was shown that an addition of at least 20% yoghurt is necessary to reduce the erosive potential.

Patients with increased erosive dental hard tissue loss should be made aware of the erosive potential of salad dressings to make an informed decision. If they do not refrain from the consumption of salad dressing, they should be encouraged to modify the dressings with plain yoghurt in order to at least reduce the erosive potential.

## References

[ref1] Affolter U (2003). Italienische Salatsauce. In: Tiptopf: Interkantonales Lehrmittel für den Hauswirtschaftsunterricht.

[ref2] Almeida e Silva JS, Baratieri LN, Araujo E, Widmer N (2011). Dental erosion: understanding this pervasive condition. J Esthet Restor Dent.

[ref3] Attin T, Becker K, Roos M, Attin R, Paqué F (2009). Impact of storage conditions on profilometry of eroded dental hard tissue. Clin Oral Investig.

[ref4] Attin T, Becker K, Wiegand A, Tauböck TT, Wegehaupt FJ (2013). Impact of laminar flow velocity of different acids on enamel calcium loss. Clin Oral Investig.

[ref5] Attin T, Buchalla W, Gollner M, Hellwig E (2000). Use of variable remineralization periods to improve the abrasion resistance of previously eroded enamel. Caries Res.

[ref6] Attin T, Wegehaupt F, Gries D, Wiegand A (2007). The potential of deciduous and permanent bovine enamel as substitute for deciduous and permanent human enamel: Erosion-abrasion experiments. J Dent.

[ref7] Aykut-Yetkiner A, Wiegand A, Ronay V, Attin R, Becker K, Attin T (2014). In vitro evaluation of the erosive potential of viscosity-modified soft acidic drinks on enamel. Clin Oral Investig.

[ref8] Barbour ME, Lussi A (2014). Erosion in relation to nutrition and the environment. Monogr Oral Sci.

[ref9] Braden M (1964). Heat conduction in normal human teeth. Arch Oral Biol.

[ref10] Chan AS, Tran TTK, Hsu YH, Liu SYS, Kroon J (2020). A systematic review of dietary acids and habits on dental erosion in adolescents. Int J Paediatr Dent.

[ref11] Christensen CM, Navazesh M (1984). Anticipatory salivary flow to the sight of different foods. Appetite.

[ref12] Eccles JD (1982). Tooth surface loss from abrasion, attrition and erosion. Dent Update.

[ref13] Eisenburger M, Addy M (2003). Influence of liquid temperature and flow rate on enamel erosion and surface softening. J Oral Rehabil.

[ref14] Esser M, Tinschert J, Marx R (1998). Materialkennwerte der Zahnhartsubstanz des Rindes im Vergleich zur humanen Zahnhartsubstanz. Dtsch Zahnärztl Z.

[ref15] Ganss C, Schlueter N, Friedrich D, Klimek J (2007). Efficacy of waiting periods and topical fluoride treatment on toothbrush abrasion of eroded enamel in situ. Caries Res.

[ref16] Hannig M (1999). Ultrastructural investigation of pellicle morphogenesis at two different intraoral sites during a 24-h period. Clin Oral Investig.

[ref17] Hannig M, Hannig C (2014). The pellicle and erosion. Monogr Oral Sci.

[ref18] Hannig M, Hess NJ, Hoth-Hannig W, De Vrese M (2003). Influence of salivary pellicle formation time on enamel demineralization –an in situ pilot study. Clin Oral Investig.

[ref19] Hannig M, Joiner A (2006). The structure, function and properties of the acquired pellicle. Monogr Oral Sci.

[ref20] Hara AT, Zero DT (2014). The potential of saliva in protecting against dental erosion. Monogr Oral Sci.

[ref21] Hartz JJ, Procopio A, Attin T, Wegehaupt FJ (2021). Erosive potential of bottled salad dressings. Oral Health Prev Dent.

[ref22] Hellwig E, Lussi A (2014). Oral hygiene products, medications and drugs –hidden aetiological factors for dental erosion. Monogr Oral Sci.

[ref23] Heurich E, Beyer M, Jandt KD, Reichert J, Herold V, Schnabelrauch M (2010). Quantification of dental erosion-a comparison of stylus profilometry and confocal laser scanning microscopy (CLSM). Dent Mater.

[ref24] Hong DW, Lin XJ, Wiegand A, Yu H (2020). Does delayed toothbrushing after the consumption of erosive foodstuffs or beverages decrease erosive tooth wear? A systematic review and meta-analysis. Clin Oral Investig.

[ref25] Hunt JN (1951). The composition of gastric juice. J Physiol.

[ref26] Körner P, Inauen DS, Attin T, Wegehaupt FJ (2020). Erosive/abrasive enamel wear while using a combination of anti-erosive toothbrush/-paste. Oral Health Prev Dent.

[ref27] Larsen MJ, Nyvad B (1999). Enamel erosion by some soft drinks and orange juices relative to their pH, buffering effect and contents of calcium phosphate. Caries Res.

[ref28] Laurance-Young P, Bozec L, Gracia L, Rees G, Lippert F, Lynch RJM (2011). A review of the structure of human and bovine dental hard tissues and their physicochemical behaviour in relation to erosive challenge and remineralisation. J Dent.

[ref29] Lee VM, Linden RW (1992). An olfactory-submandibular salivary reflex in humans. Exp Physiol.

[ref30] Lussi A, Carvalho TS (2014). Erosive tooth wear: a multifactorial condition of growing concern and increasing knowledge. Monogr Oral Sci.

[ref31] Lussi A, Hellwig E (2014). Risk assessment and causal preventive measures. Monogr Oral Sci.

[ref32] Lussi A, Hellwig E, Ganss C, Jaeggi T (2009). Buonocore Memorial Lecture Dental erosion. Oper Dent.

[ref33] Lussi A, Jaeggi T, Zero D (2004). The role of diet in the aetiology of dental erosion. Caries Res.

[ref34] Lussi A, Schaffner M (2000). Progression of and risk factors for dental erosion and wedge-shaped defects over a 6-year period. Caries Res.

[ref35] Mellberg JR (1992). Hard-tissue substrates for evaluation of cariogenic and anti-cariogenic activity in situ. J Dent Res.

[ref36] Moazzez R, Bartlett D (2014). Intrinsic causes of erosion. Monogr Oral Sci.

[ref37] O’Sullivan EA, Curzon ME (2000). A comparison of acidic dietary factors in children with and without dental erosion. ASDC J Dent Child.

[ref38] Pace F, Pallotta S, Tonini M, Vakil N, Bianchi Porro G (2008). Systematic review: gastro-oesophageal reflux disease and dental lesions. Aliment Pharmacol Ther.

[ref39] Saads Carvalho T, Lussi A (2020). Chapter 9: Acidic beverages and foods associated with dental erosion and erosive tooth wear. Monogr Oral Sci.

[ref40] Salas MM, Nascimento GG, Vargas-Ferreira F, Tarquinio SB, Huysmans MC, Demarco FF (2015). Diet influenced tooth erosion prevalence in children and adolescents: Results of a meta-analysis and meta-regression. J Dent.

[ref41] Schlueter N, Hara A, Shellis RP, Ganss C (2011). Methods for the measurement and characterization of erosion in enamel and dentine. Caries Res.

[ref42] Shellis RP, Featherstone JD, Lussi A (2014). Understanding the chemistry of dental erosion. Monogr Oral Sci.

[ref43] Shellis RP, Finke M, Eisenburger M, Parker DM, Addy M (2005). Relationship between enamel erosion and liquid flow rate. Eur J Oral Sci.

[ref44] Siqueira WL, Custodio W, McDonald EE (2012). New insights into the composition and functions of the acquired enamel pellicle. J Dent Res.

[ref45] Steiger-Ronay V, Steingruber A, Becker K, Aykut-Yetkiner A, Wiedemeier DB, Attin T (2018). Temperature-dependent erosivity of drinks in a model simulating oral fluid dynamics. J Dent.

[ref46] Steiger-Ronay V, Stelz S, Steigmeier D, Becker K, Wiedemeier DB, Attin T (2019). Change of erosive potential of apple and orange juice at different dilutions. Swiss Dent J.

[ref47] Steiger-Ronay V, Tektas S, Attin T, Lussi A, Becker K, Wiedemeier DB (2019). Comparison of profilometric and microindentation analyses for determining the impact of saliva on the abrasion of initially eroded enamel. Caries Res.

[ref48] ten Cate JM, Imfeld T (1996). Dental erosion, summary. Eur J Oral Sci.

[ref49] Wang X, Lussi A (2010). Assessment and management of dental erosion. Dent Clin North Am.

[ref50] Wegehaupt F, Günthart N, Sener B, Attin T (2011). Prevention of erosive/abrasive enamel wear due to orange juice modified with dietary supplements. Oral Dis.

[ref51] Wegehaupt FJ, Lunghi N, Hogger VM, Attin T (2016). Erosive potential of vitamin and vitamin+mineral effervescent tablets. Swiss Dent J.

[ref52] Wiegand A, Attin T (2011). Design of erosion/abrasion studies-insights and rational concepts. Caries Res.

[ref53] Wiegand A, Stock A, Attin R, Werner C, Attin T (2007). Impact of the acid flow rate on dentin erosion. J Dent.

[ref54] Yassen GH, Platt JA, Hara AT (2011). Bovine teeth as substitute for human teeth in dental research: a review of literature. J Oral Sci.

[ref55] Zero DT (1996). Etiology of dental erosion-extrinsic factors. Eur J Oral Sci.

[ref56] Zimmer S, Kirchner G, Bizhang M, Benedix M (2015). Influence of various acidic beverages on tooth erosion. Evaluation by a new method. PLoS One.

[ref57] Zipkin I, McClure FJ (1949). Salivary citrate and dental erosion; procedure for determining citric acid in saliva; dental erosion and citric acid in saliva. J Dent Res.

